# Development of Highly Crystalline Polylactic Acid with β-Crystalline Phase from the Induced Alignment of Electrospun Fibers

**DOI:** 10.3390/polym13172860

**Published:** 2021-08-25

**Authors:** Coro Echeverría, Irene Limón, Alexandra Muñoz-Bonilla, Marta Fernández-García, Daniel López

**Affiliations:** 1 Instituto de Ciencia y Tecnología de Polímeros (ICTP-CSIC), C/Juan de la Cierva 3, 28006 Madrid, Spain; irenelimonvelo@gmail.com (I.L.); sbonilla@ictp.csic.es (A.M.-B.); martafg@ictp.csic.es (M.F.-G.); 2 Interdisciplinary Platform for Sustainable Plastics towards a Circular Economy-Spanish National Research Council (SusPlast-CSIC), 28006 Madrid, Spain

**Keywords:** polylactic acid, electrospinning, β-crystalline phase

## Abstract

Polylactic acid (PLA) is one of the known synthetic polymers with potential piezoelectric activity but this property is directly related to both the crystalline structure and crystalline degree. Depending on the process conditions, PLA can crystallize in three different forms: α-, β-, and γ- form, with β-crystalline phase being the piezoelectric one. To obtain this crystalline structure, transformation of α to β is required. To do so, the strategies followed so far consisted in annealing or/and stretching of previously obtained PLA in the form of films or fibers, that is, additional post-processing steps. In this work, we are able to obtain PLA fibers with high macromolecular alignment, as demonstrated by SEM, and in the β polymorph, as detected by X-ray diffraction (XRD) without the requirement of post-processing. For that, PLA fibers were prepared by using an electrospinning coupled to a drum collector. This set up and the optimization of the parameters (voltage flow-rate, and drum collector speed) induced molecular stretching giving rise to uniaxially oriented and highly aligned fibers.

## 1. Introduction

Currently, society is facing one of its greatest challenges to reduce the global dependence on fossil fuel sources. One of the most direct strategies to face this challenge is the development of sustainable and eco-friendly functional materials from natural or bio-based and biodegradable polymers or biopolymers, which are able to replace non-degradable synthetic polymers. One of the biopolymers in which more research effort is being invested is polylactic acid (PLA) due to its applicability in different fields of science and technology [1,2,3]. PLA, besides of being biodegradable, biocompatible and low toxic, is one of the three known synthetic polymers (together with polyvinylidene fluoride PVDF and polyhydroxybutyrate PHB) with potential piezoelectric activity, which is the capacity to produce an electrical charge in response to an applied mechanical stress and vice-versa [4]. Among the three polymers, PVDF and its copolymers have been widely studied. Their high piezoelectric response is increasingly being used in an extensive range of technological applications, such as energy generation and storage [5], monitoring and control [6], biomedicine [7], sensors and actuators [8], and smart scaffolds [9,10]. In particular, piezoelectric materials applications involving interfaces with biological systems represent an exciting area of rapid development [11,12]. For instance, Han et al., created 3D piezoelectric systems for energy harvesting and biomedical implants based on PVDF thin-films, which were implanted in the leg muscle of a rat to evaluate the energy generated by the rat movement [13]. Despite the fact that PVDF is biocompatible, the main drawback of this polymer is its fossil-fuel origin besides its lack of biodegradability, with the subsequent impact on the environment. Therefore, and as mentioned above, to contribute to the circular economy of plastics and support the development of sustainable polymers with advanced applications much effort has been invested in PLA biopolymer with a wide range of potential biomedical applications as well as in the sector of so-called “wearables” [14,15,16,17].

PLA is a linear polyester that has two stereoisomers: poly(L-lactic acid) (PLLA), poly(D-lactic acid) (PDLA), both semi-crystalline and, the racemic mixture PDLLA, which is amorphous. PLA properties, crystallinity, melting temperature and the mechanical properties are directly affected by the polymer structure, which is also dependent on the L to D ratio. As a matter of fact, it was determined that for each 1% of D-isomer in PLA, a decrease of 5 °C in the melting temperature was observed [18].

As known, PLA is formed by chiral molecules where –COO– polar groups are bonded to an asymmetric carbon and oriented in helical conformation. Depending on the process conditions, PLA can crystallize in three different forms: α-, β-, and γ- form; being α-crystalline phase the most stable and β-phase the one that provides piezoelectric characteristics. The α polymorph is characterized by a 10_3_ helicoidal conformation (having 10 helix repeat structures in three chain twists) packed in an orthorhombic unit cell, and is obtained from the crystallization of the melt or from PLA solutions. The β-crystalline form is characterized by a 3_1_ helical structure packed in a triclinic cell unit. Although the α-crystalline form in PLA is the most thermodynamically stable, this conformation of PLA is not piezoelectric due to its non-polarity, caused by the orientation of C=O dipoles in all directions along the main chain. Thus, the transformation from α- to β-form is required in order to obtain piezoelectric response. For instance, in a recent work Oumghar et al., incorporated different amounts of graphene oxide nanosheets into PLA films to induce the development of the β polymorph and, therefore, enhance the piezoelectric property [19]. However, the mostly used strategy to obtain the β polymorph involves the application of a stress-induced post-processing step. In this sense, PLA that crystallized in the α-phase, is stretched and/or annealed (hot-drawing at different drawing rations). When α-conformation of PLA is stretched (sheared) through its side chain, the –C=O dipole makes a slight rotation that changes the polarization of the chain molecule. As a consequence, polarization appears in a direction perpendicular to the plane of applied stress. Therefore, this processing provokes the transformation into β polymorph, in order to induce the required molecular orientation besides increasing the degree of crystallinity [20,21,22,23].

Up to the best of our knowledge, there are research works published dealing with the seeking of optimal electrospinning parameters to obtain uniaxially oriented PLA fibers or directly studies based on aligned PLA electrospun fibers for biomedical applications [14,24,25]. There are also studies based on the development of the β-crystalline phase on PLA films and very few on electrospun fibers, but with the required posterior drawing and/or annealing step as already mentioned [21,22,23]. Our intention with this work is to eliminate the need for such a post-processing step in order to facilitate the scale-up of this material, which might be industrially relevant. We aim to develop a one-step PLA processing that could result directly in highly oriented molecular chains crystallized in the β-form. To do so, we process PLA through electrospinning coupled to a rotatory drum collector, capable of forming polymer fibers with diameters ranging from micrometer to nanometer scales with high surface-to-volume ratio, flexibility, tunable porosity [26,27] and, moreover, able to induce fiber alignment [28,29]. The resultant fibers can be used in a variety of applications, ranging from sensors, drug-delivery systems, tissue regeneration, among others [30,31,32,33,34,35]. In the case of PLA, certain electrospinning conditions and set-ups, such as the coupling of rotatory cylinder (drum) as fiber collector, contribute to the stretching of the polymer while collecting the fibers in the drum. This could lead to macroscopically aligned and simultaneously stretched fibers that could induce molecular orientation within the polymer chain in a single step [23,36]. Therefore, the purpose of this work is to take advantage of the electrospinning technique to optimize the process and obtain most suitable parameters. This procedure will end up in highly aligned and oriented PLA electrospun fibers crystallized in the β polymorph without the requirement of post-processing, which has been the strategy followed so far. For that, we have performed an extensive study of the electrospinning parameters and PLA polymer solution parameters to find the suitable conditions. The outcome of this work will be the first step for the development of a bio-based and sustainable polymer that would have potential applications as an electroactive polymer resulting in piezoelectric actuators [17].

## 2. Materials and Methods

### 2.1. Materials

Biodegradable poly(lactic acid) (PLA) was supplied by NatureWorks^®^ (Minnetonka, MN, USA) under the trade name PLA6202D, with >2% of D-lactic acid monomer and a weight-average molecular weight (M_w_) of 157.5 kDa. Chloroform (CHCl_3_) and N,N-dimethylformamide (DMF) purchased from Sigma Aldrich (Madrid, Spain) were used as solvents.

### 2.2. Solution Preparation

PLA was purified before use by dissolving in chloroform, precipitation in ethanol and further drying under vacuum during 24 h to eliminate any solvent. For the electrospinning solution, different concentrations of PLA, in the range of 10 to 20 wt.%, were dissolved in CHCl_3_:DMF solvent systems using two solvent ratios: 80:20 and 90:10. The samples were kept under magnetic stirring for 24 h at room temperature until the complete dissolution of PLA.

### 2.3. Electrospinning

**Figure 1:**
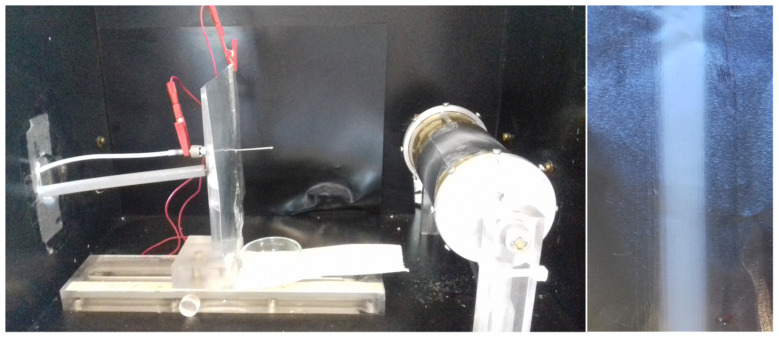
Photographs of the home-made electrospinner equipped used in this study (**left**) and of the one example of the obtained electrospun polymer fibers (**right**).

Electrospun polymeric fibers were prepared using a home-made electrospinner equipped with a syringe needle (blunt type 0.584 mm inner diameter) connected to a high voltage power supply (Spellman SL Serie 30 kV 600 W) with horizontal configuration (Figure 1). The as-prepared solutions were transferred into a 5 mL syringe and loaded into a syringe pump (KDS100, Kd Scientific) placed parallel to the ground and programmed to deliver the solution at constant flow. To promote the alignment of the fibers, we used a cylindrical collector (diameter of 7 cm) rotating at different speeds. The electrospinning process was carried out at room temperature and the relative humidity was maintained between 30% and 40%. The obtained non-woven mats were vacuum dried at room temperature for 24 h to remove any solvent residues. For the sake of comparison, random PLA electrospun fibers, 20 wt.% and solvent ratio of 90:10 [CHCl_3_:DMF] were prepared using a static plate as collector, thus without inducing any stretching.

Different electrospinning conditions were tested to establish the optimal electrospinning parameters necessary to obtain highly aligned non-woven fibers mats with narrow diameter size distribution and free of defects. The conditions used in this work are collected in Table 1**.**

### 2.4. Characterization Methods

The viscosity of PLA fiber precursor solutions used was determined by means of an AR1000 rheometer (TA Instruments). Steady state flow curves were performed at 5 °C to avoid solvent evaporation issues, with 40 mm diameter cone-plate steel geometry.

The morphology of the PLA electrospun fibers was studied using scanning electron microscope (SEM) Philips XL30 with an acceleration voltage of 25 kV. The electrospun fibers were sputter coated with gold prior to scanning. The orientation and diameter size were determined from SEM images using Image J software.

For the structural characterization of the obtained fibers a Bruker D8 ADVANCE diffractometer X-ray diffractometer (XRD) was used. The measurements were carried out using Cu Kα radiation (λ = 1.54 Å) in the range of 2θ = 2°–70° and a scan velocity of 0.5 °/min. Electrospun fiber mats were analyzed by differential scanning calorimetry (DSC) using the TAQ2000 calorimeter from TA instruments. For this characterization, the samples were separated from the aluminum foil and cut into several pieces, from the middle region of the electrospun mat. These pieces were placed layer by layer into the aluminum pan in order to reach a minimum weight of 1 mg. Each sample was put through a single temperature sweep from −80 °C to 200 °C at a heating rate of 10 °C/min, and under nitrogen gas flow. From the DSC experiments the glass transition temperature (Tg), melting temperature (Tm) and cold crystallization temperature (Tcc) were determined. In addition to this, the percentage of crystallinity (Xc) was calculated as determined in Equation (1), subtracting the enthalpy of crystallization (∆Hcrystallization) from the enthalpy of fusion (∆Hfusion) and dividing it by the enthalpy fusion corresponding to a single PLA crystal (∆H fusion, perfect crystal = 93.6 J/g) [37].

(1) $$\%\;Cristallinity\;\left(Xc\right)=\left(\frac{\Delta H_{fusion}-\Delta H_{cystallization}}{\Delta H_{fusion,\;PLA\;perfect\;crystal}}\right)\times 100$$

To evaluate the thermal stability, thermogravimetric analysis (TGA) was performed on a TA Instruments (TGA Q500, TA instruments) at a heating rate of 10 °C/min from 25 to 800 °C, under nitrogen atmosphere.

The mechanical properties of the samples analyzed using an Instron universal testing machine. Small rectangular pieces of PLA electrospun non-woven mat (2 cm × 0.4 cm) were cut in two orthogonal directions with the longest dimension of the sample parallel to the direction of the fiber. The samples were stretched uniaxially at a constant rate of 1 mm/min along the longest sample dimension at room temperature. The values of the mechanical properties of a given sample were taken to be the average of the results of five successful measurements.

## 3. Results and Discussion

### 3.1. Optimization of Electrospinning Process

#### 3.1.1. Effect of Polymer Solution Parameters: Polylactic Acid (PLA) Concentration and CHCl_3_:DMF Ratio

**Figure 2:**
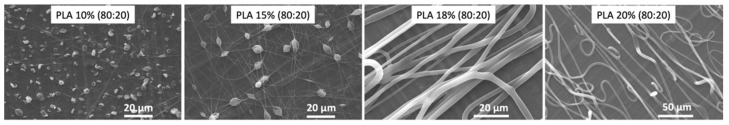
Scanning electron microscope (SEM) images corresponding to polylactic acid (PLA) fibers obtained with different PLA concentration (10, 15, 18 and 20 wt.%) and fixed solvent system (80:20 CHCl_3_:DMF) and electrospinning parameters (16 kV, feeding rate of 1.0 mL/h and rotor velocity of 1300 rpm).

Regarding the effect of PLA concentration, Figure 2 depicts representative SEM micrographs of PLA fibers obtained from fiber precursor solutions containing 10, 15, 18 and 20 wt.% of PLA and a solvent ratio of 80:20 CHCl_3_:DMF. As observed from the micrographs, the use of polymer solutions containing either 10 or 15 wt.% of PLA gives rise to electrospun fibers with defects (beads). In contrast, electrospun fibers obtained from solutions with 18 and 20 wt.% of PLA are defect-free. This could be due to the viscosity of the fiber precursor solutions as shown in Table 2.

If the viscosity of the solution is too low, the dominant factor is the surface tension of the droplet, which results in the inability of forming a continuous fiber and mainly drops of solution or beaded fibers reaching the collector [38]. This is the case for polymer solutions containing either 10 or 15 wt.% of PLA. On the other hand, if the viscosity of the solution is too high, the electric field may not be enough to disrupt the viscoelastic forces, which could lead to the formation of fibers with irregular diameters or even cause drying polymer solution at the needle tip [39].

**Figure 3:**
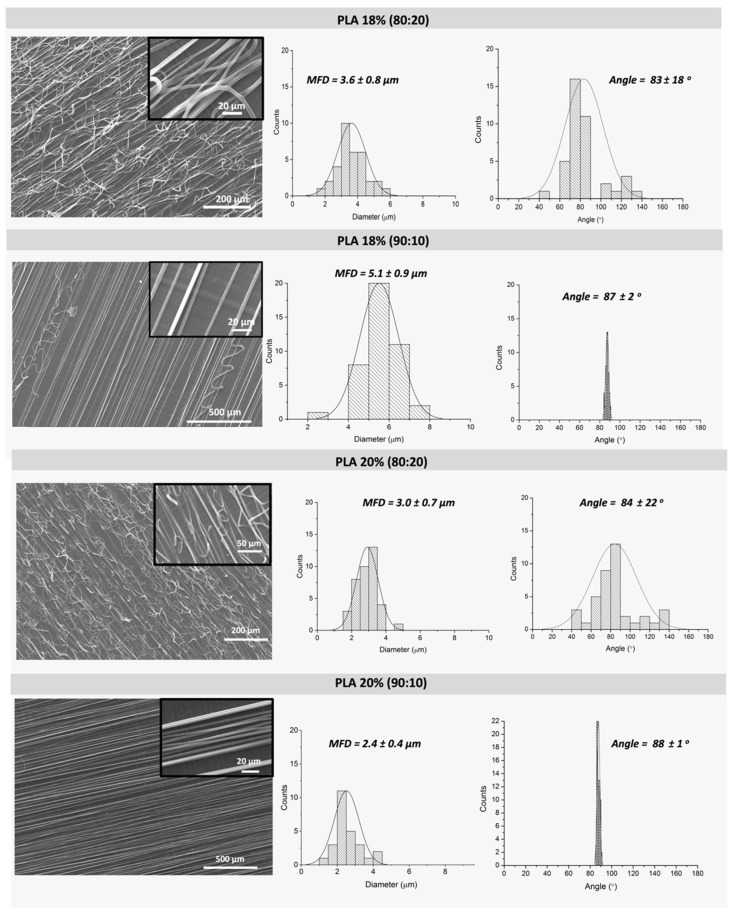
SEM micrographs corresponding to PLA fibers obtained with different PLA concentrations of 18 and 20 wt.% and CHCl_3_:DMF with different ratios 80:20 and 90:10, with their corresponding histograms to determine mean fiber diameter (MFD) and orientation. (Electrospinning fixed parameters: 16 kV, needle-to-collector distance 12 cm, feeding rate of 1.0 mL/h and rotor velocity of 1300 rpm).

By using a fixed concentration of PLA, 18 and 20 wt.%, we evaluated the influence of CHCl_3_:DMF solvent ratios (80:20 and 90:10) in the fiber morphology: mean fiber diameter (MFD) and orientation/alignment. As observed from Figure 3, the increase of DMF content gives rise to fibers more susceptible to fusion (see insets of Figure 3). This effect is not observed for the lowest content of DMF, CHCl_3_:DMF solvent ratio of 90:10. DMF contributes to increase the surface charge of the spinning jet, which leads to stronger elongation forces and thus, defect-free fibers. However, the increase of DMF content also affects the volatility of the solution. The ejected fibers reach the collector without being completely dry as the solvent has not been completely removed, thus causing fusion points between fibers [40,41].

The use of a CHCl_3_:DMF solvent ratio of 90:10 gave rise to fibers with no fusion points among them, and what is more relevant, this resulted in the formation of highly aligned fibers as observed in Figure 3.

By evaluating the effect of PLA concentration in the fibers morphology obtained using CHCl_3_:DMF solvent ratio of 90:10, we could concluded that 20 wt.% PLA fibers (see Figure 3) showed lower mean fiber diameter (MFD) and higher degree of alignment with a narrower average orientation compared to 18 wt.% PLA fibers. This first analysis allowed to stablish that the most suitable solution parameters to obtain defect-free, and oriented fibers are 20 wt.% PLA and CHCl_3_:DMF ratio of 90:10.

#### 3.1.2. Effect of Collector Speed

**Figure 4:**
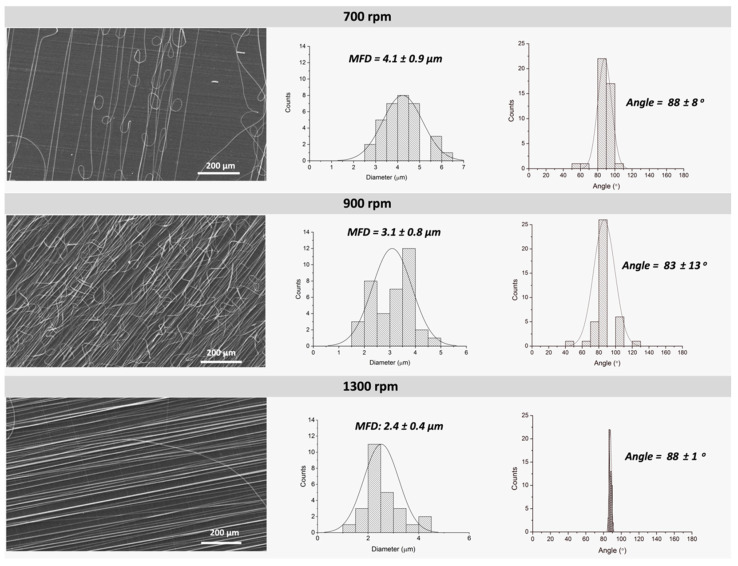
SEM micrographs of PLA fibers (20 wt.% PLA, 90:10 CHCl_3_:DMF) with their respective histograms corresponding to mean fiber diameter (MFD) and orientation as a function of rotor velocity. (Electrospinning fixed parameters: 16 kV, needle-to-collector distance 12 cm and feeding rate of 1.0 mL/h).

In order to optimize the electrospun fiber orientation and mean fiber diameter (MFD), different rotor speeds were tested. Figure 4 shows representative SEM micrographs of PLA fibers (20 wt.% PLA and 90:10 CHCl_3_:DMF ratio) collected at three different rotor speeds: 700, 900 and 1300 rpm. As observed from the micrographs and represented in the corresponding histograms, fibers obtained at 700 and 900 rpm exhibited less aligned fibers but showing certain preferential orientation. In the case of fibers collected at the highest rotation speed (1300 rpm), highly oriented fibers aligned in one preferential direction can be observed. Similar results were already described for PLA fibers studied by Ribeiro et al. [24] where they could estimate what they called the alignment speed, defined as the matching of linear speed of the rotating fiber collector surface with the speed of the exerted jet when deposits. At that speed, the fibers are collected circumferentially at the surface of the cylindrical collector ending up in highly aligned fibers. In the case of PLA6202D, we could determine that the alignment speed was 1300 rpm as demonstrated by SEM micrographs and their respective histograms.

In addition to the fiber alignment, the increase of rotation speed also affects the diameter of the electrospun fibers. As can be seen from the histograms (determined from SEM micrographs), as the rotary speed increases the mean fiber diameter decreases showing a narrower curve and thus, resulting in more homogeneous fibers.

#### 3.1.3. Effect of Feeding Rate

**Figure 5:**
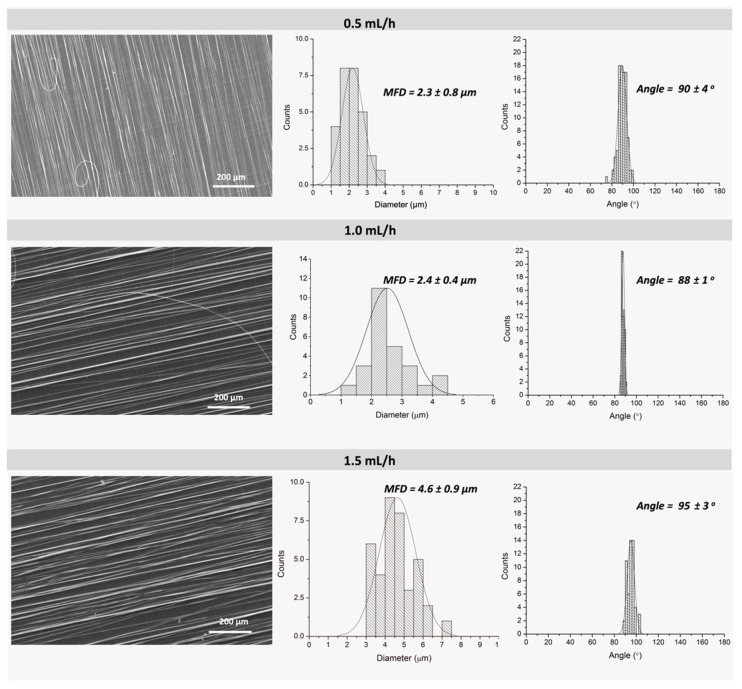
SEM micrographs (row 1) corresponding to PLA fibers (20 wt.% PLA, 90:10 CHCl_3_:DMF) obtained using 3 different feeding rates: 0.5, 1.0 and 1.5 mL/h and their respective histograms corresponding to mean fiber diameter and orientation. (Electrospinning fixed parameters: 16 kV, needle-to-collector distance 12 cm and rotor velocity 1300 rpm).

Figure 5 shows representative SEM images corresponding to PLA fibers electrospun at a constant feeding rate of 0.5, 1.0 and 1.5 mL/h. When analyzing the histograms corresponding to MFD, an expected increase of fiber diameter is observed with the increase of solution feed rate, going from 2.3–2.4 µm to 4.6 µm for solution feed rate of 0.5–1.0 mL/h and 1.5 mL/h, respectively. The increase of fiber diameter is directly related to the fact that higher feed rate provides more polymer solution (volume) to the tip of the needle. However, there are no significant differences in MFD, whereas only slight differences in the orientation were observed when comparing feeding rates of 0.5 and 1.0 mL/h. Nevertheless, the use of a low feed rate of 0.5 mL/h required a longer electrospinning time to obtain non-woven mats with the necessary thickness for the subsequent characterization. This longer time resulted in evaporation of the solvent in both the syringe and the needle, leading to blockage. Therefore, these results and experimental observations led us to select the feeding rate of 1.0 mL/h as most suitable.

#### 3.1.4. Effect of Voltage

**Figure 6:**
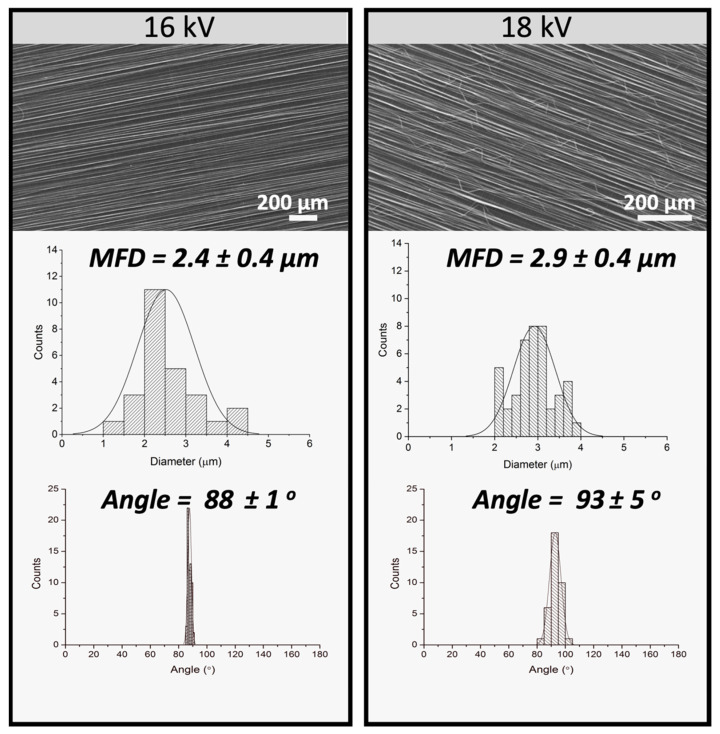
SEM micrographs (row 1) corresponding to PLA fibers (20% PLA, 90:10 CHCl_3_:DMF) obtained applying different voltages: 16, 18 kV, and their respective histograms corresponding to mean fiber diameter (row 2) and orientation (row 3). (Electrospinning fixed parameters: 1.0 mL/h feed rate, needle-to-collector distance 12 cm and rotor velocity 1300 rpm).

Figure 6 shows SEM micrographs, MFD and orientation histograms corresponding to PLA fibers (PLA 20 wt.%; CHCl_3_:DMF 90:10; feed rate of 1.0 mL/h, 1300 rpm) with variable applied voltage: 16 and 18 kV. The variation of the voltage was proposed as a method to obtain smaller MFD [40]. In this case, the increase in voltage did not affect fiber diameter, but affected negatively the orientation of the fibers. This effect could be due to the fact that the increase in the applied voltage can lead to a faster acceleration/projection of the solution when is exerted from the needle towards the collector. This faster acceleration may cause the formation of secondary jets, providing some instability in the deposition of the fibers on the collector.

From this morphological analysis it was concluded that (i) the most suitable solution parameters corresponded to 20 wt.% PLA and CHCl_3_:DMF ratio of 90:10 and that (ii) the most suitable electrospinning conditions were: rotation speed of 1300 rpm, feed rate of 1.0 mL/h, and a voltage of 16 kV, with a fixed distance of 12 cm between the needle and the collector. These parameters resulted in defect-free, homogeneous and highly aligned PLA fibers.

In order to evaluate the influence of the fiber structure obtained, orientation and alignment, the following two samples, prepared under the electrospinning conditions described before, were selected for further characterization: (1) 20 wt.% PLA and CHCl_3_:DMF ratio of 80:20, that give rise to PLA fibers with a preferential orientation (but not highly aligned), from now on labeled as: po-PLA and, (2) 20 wt.% PLA and CHCl_3_:DMF ratio of 90:10 resulting in highly aligned PLA fibers from now on designated as: ha-PLA.

### 3.2. Effect of Fiber Alignment/Orientation in the Crystalline form of PLA

As described in the introduction, different crystal phases or structures have been reported for PLA, which depend on the crystallization conditions. The most stable crystalline structure is the α-form, which occurs in conventional melt and solution crystallization conditions. PLA can also crystallize forming a β-phase, which has also been described as the polymorph required to provide piezoelectric activity to PLA [42].

**Figure 7:**
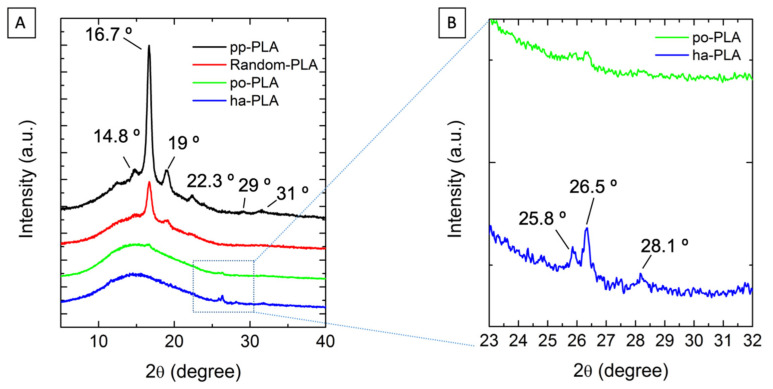
(**A**) X ray diffractograms corresponding to pp-PLA, random-PLA, po-PLA and ha-PLA (highly oriented) fibers. (**B**) Inset of the scale (2θ = 23 to 32°) corresponding to PLA β-form related region.

To evaluate the effect that stretching and fiber alignment could have in the crystalline structure developed during the electrospinning process, we performed X-ray diffraction experiments to purified powder PLA (named as pp-PLA), po-PLA fibers and ha-PLA fibers as depicted in Figure 7A. As observed from the figure, pp-PLA showed the characteristic diffraction peaks corresponding to the α-form: 2θ = 14.5° (010), 16.7° (200)/(110), 19°, 29° and 31° (0010). However, in the po-PLA and ha-PLA fibers diffraction patterns, new peaks appeared in the range of 2θ ≈ 23–32° while diffraction peaks corresponding to the α structure are not clearly observed; only in the case of po-PLA a very weak diffraction peak is shown at 2θ = 16.7° (200). In Figure 7B, which is the amplification of the 2θ ≈ 23–32° range, it is clearly observed that these new diffraction peaks appearing at 2θ ≈ 25.8 and 26.3° for po-PLA and at 2θ ≈ 25.8, 26.3 and 28.1° for ha-PLA, corresponding to the β crystalline form [43].

As stated in the literature, such crystalline form can be created by stretching PLA (films or fibers) at high draw-ratio and/or annealing at high temperature, that is, by applying a post-processing stage [22,44]. Therefore, this result confirms that the electrospinning technique (at certain suitable conditions) besides inducing the alignment of the fibers during the collection in the rotatory cylinder, also stretches the fiber and thus transforms the most thermodynamically stable crystalline form into the “piezoelectric” β-crystalline phase [36,45]. It is worth noting that in the case of ha-PLA the diffraction peaks are more intense than in po-PLA (see Figure 7B). This could probably be due to a higher molecular orientation of the fibers during stretching, which also caused an increase in crystallinity as further determined from DSC measurements.

### 3.3. Thermal Properties and Crystallization of PLA Fibers

**Figure 8:**
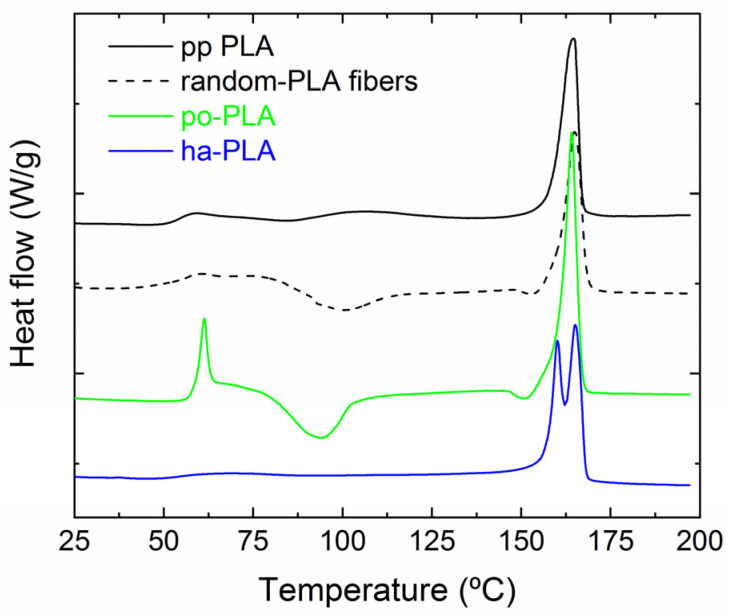
Differential scanning calorimetry (DSC) heating curves of pp-PLA, random-PLA fibers, partially oriented (po-PLA) and highly oriented fibers (ha-PLA) at a heating rate of 10 °C/min.

We also evaluated the effect of fiber arrangement on the crystallization and thermal properties of the selected PLA fibers by means of DSC. For the sake of comparison, we also performed the test on random PLA fibers obtained using a static plate as collector. In Figure 8 DSC heating curves of pp-PLA, random-PLA, po-PLA and ha-PLA fiber mats are shown. The corresponding results are collected in Table 3, where glass transition temperature (Tg), cold crystallization temperature (Tcc), melting temperature (Tm) and Xc (calculated from Equation (1)) are summarized. As can be seen from Figure 8, random PLA fibers (prepared without rotational drum) as well as po-PLA fibers (with preferentially oriented fibers) showed cold crystallization peaks at 101 °C and 94 °C, respectively. Cold crystallization is a phenomenon that occurs when the sample is heated from the amorphous solid state, instead of occurring when cooling from the melting state, which leads to the formation of crystals [18]. As demonstrated, the electrospinning technique enhances polymer chain orientation but produces an incomplete crystallization of the material, that is, a metastable crystalline state in which non-crystalline polymer chains are highly oriented [46]. This non-equilibrium chain conformations further crystallized when annealed as described by Ribeiro et al. [24]. However, when analyzing the DSC curve corresponding to ha-PLA fibers no cold crystallization is observed. This result clearly indicates that a complete crystallization was reached during the electrospinning process under the selected conditions probably due to the molecular stretching of the polymer. In addition, it is worth highlighting the presence of two melting peaks in the DSC curve of ha-PLA fibers at 160 and 165 °C, respectively. Considering the results obtained from X-ray diffraction analysis, these two melting peaks could be related to the formation of two crystalline polymorphs: β crystalline form (Tm ≈ 160 °C) and α crystalline form (Tm ≈ 165 °C).

We also determined the Xc (see Table 3) from the DSC curves (Figure 8). As observed from the table, there is a loss of crystallinity for random-PLA (13%) and po-PLA (18%) with respect to pp-PLA (46%) that is recovered in the case of ha-PLA reaching a crystallinity of the 43%, similar to that of pp-PLA. The decrease in crystallinity observed for po-PLA fiber mat and the cold crystallization process could be due to the organization of the polymer chains as oriented amorphous regions but with the presence of numerous crystal nuclei [24]. In contrast, the electrospinning conditions applied for ha-PLA, probably enhanced the stretching of the fibers giving rise to a molecular ordering and a fiber mat with increased crystallinity [18].

**Figure 9:**
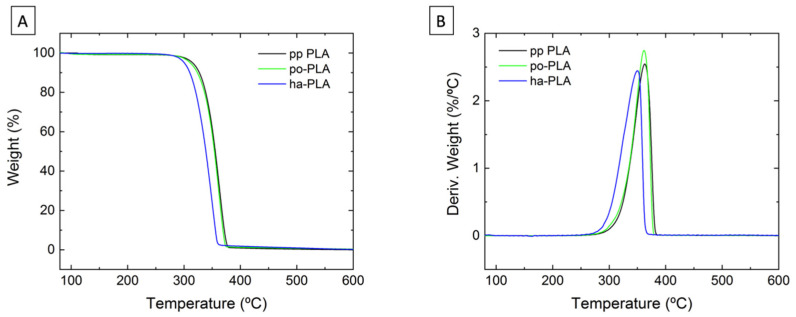
(**A**) Thermogravimetric analysis (TGA) thermograms of pp-PLA po-PLA and ha-PLA fibers at a heating rate of 10 °C/min performed under nitrogen atmosphere; (**B**) derivatives of TGA thermograms.

We also studied the influence of alignment/orientation of PLA fibers on their thermal stability. For that, thermal decomposition of pp-PLA and selected PLA fiber mats was studied by means of TGA as shown in Figure 9 with the obtained results collected in Table 4. As expected, PLA degraded in a single step process [38]. No differences were observed when comparing pp-PLA and po-PLA thermograms. However, in the case of ha-PLA fiber mat the degradation occurred at T_onset_ = 318 °C, which is 20 °C below the pp-PLA and po-PLA (T_onset_ = 336 °C). Considering that PLA used for the preparation of the ha-fibers is the same as pp-PLA and po-PLA, the difference in the onset temperature could be due to the fact that ha-PLA crystallized in the β-form, which is a thermodynamically less stable crystalline structure than the α-form as stated in the literature [18,47]. This is also evidenced in the Tm temperature obtained from the analysis of the DSC curves (Figure 8). Therefore, this result could be also considered as an indirect confirmation of the development of the β-crystalline form only using suitable electrospinning conditions and without the need for a post-processing step.

### 3.4. Evaluation of the Mechanical Properties

**Figure 10:**
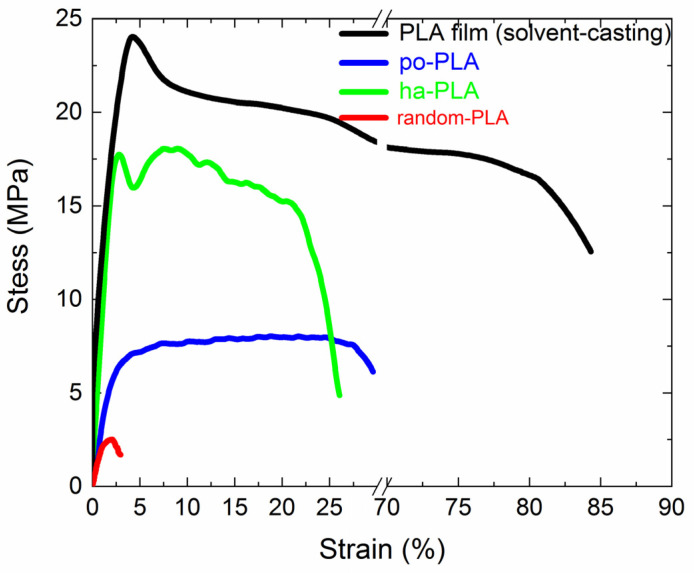
Stress-strain curves corresponding to PLA solvent-casted film (black line), partially oriented PLA electrospun fibers (blue line) and highly aligned PLA fibers (green line), and random-PLA fibers (red line).

Mechanical properties such as tensile strength, elongation at break and Young’s modulus are important parameters for the future performance of PLA electrospun fibers, for instance, as stimuli-responsive actuators as previously mentioned. Therefore, we evaluated the mechanical properties of the obtained electrospun fibers in order to determine how the uniaxial alignment of PLA fibers could affect such properties. For that, in Figure 10 representative stress-strain curves corresponding to po-PLA and ha-PLA are shown. For the sake of comparison, PLA films obtained through the solvent-casting method and random-PLA fibers were also tested and plotted. The mechanical parameters extracted from the plots are collected in Table 5. As expected, PLA film mechanical properties showed the highest values of Young’s modulus, tensile strength and elongation at break, while random PLA exhibited the lowest. When comparing the results regarding po-PLA and ha-PLA fibers, the impact of the uniaxial alignment is clearly evidenced; ha-PLA fibers Young’s modulus resulted in almost one order of magnitude higher than po-PLA and tensile strength almost three times higher. Furthermore, ha-PLA fibers’ tensile strength is similar to PLA film, within the experimental error.

In the light of the results obtained, it is worth mentioning that the uniaxial alignment of PLA fibers obtained only through specific electrospinning conditions and without any post-processing, gives rise to improved mechanical properties almost reaching those of PLA film, but without the need for additional reinforcement in the form of nanoparticles or similar.

## 4. Conclusions

In this work, we report the straightforward fabrication of PLA electrospun fibers that present the β crystalline phase and potential piezoelectricity directly from the electrospinning process without any further post-processing treatment. By taking advantage of the versatile electrospinning technique highly aligned and oriented PLA electrospun fibers were obtained by changing solution properties and process parameters such as applied voltage, feeding flow rate and collector speed. The optimal fiber alignment was observed for specific experimental conditions, 20 wt.% PLA and CHCl_3_:DMF (90:10) solution electrospun at 1300 rpm, with an applied potential of 16 kV and a feeding flow rate of 1 mL/h. Under these conditions, the stretching and rapid evaporation of the solvent induced a direct transformation of part of α crystalline structure (helical 10_3_) into β crystalline phase (helical 3_1_) as determined by XRD. This transformation was achieved without the requirement of any further treatment or post-processing step, which was the strategy followed so far. Additionally, the obtained highly aligned and oriented PLA fibers with β crystalline phase exhibited an elevated degree of crystallinity (40–50%) and enhanced mechanical properties. The impact of the uniaxial alignment was clearly evidenced in ha-PLA fibers Young’s modulus and tensile strength, which resulted in almost one order of magnitude higher and three times higher than po-PLA, respectively. The outcome of this work will be our first step in the development of bio-based and sustainable polymers with piezoelectric properties that would have potential biomedical applications as electroactive polymers for the development of actuators that can be used as skin tissue regeneration devices.

## Figures and Tables

**Table 1:** List of electrospinning parameters varied in the optimization test.

Parameter	Variation Range
PLA (wt.%)	10; 15; 18; 20
[CHCl_3_:DMF] (*v/v*)	[80:20]; [90:10]
Flow rate of syringe pump (mL/h)	0.5; 1.0; 1.5
Distance (cm)	12
Voltage (kV)	16; 18
Rotation speed of the collector (rpm)	700; 900; 1300

**Table 2:** Viscosity of the different PLA solutions tested.

Concentration	10% [80:20]	15% [80:20]	18% [80:20]	20% [80:20]	18% [90:10]	20% [90:10]
Viscosity (at shear rate 1 s^−1^) (Pa.s)	1.6	9.4	15.4	47.6	70.6	100.1

**Table 3:** Temperatures of glass transition (T_g_), cold crystallization (T_cc_), melting (T_m_) and crystallinity percentage Xc of samples. Errors in T ± 1 °C.

Samples	T_g_	T_cc_	T_m_		Xc
	(°C)	(°C)	(°C)		(%)
pp-PLA	54	-	164		46
random-PLA	56	101	164		13
po-PLA	58	94	164		18
ha-PLA	55	-	160	165	43

**Table 4:** Degradation temperatures of onset and maximum peak of samples in an inert atmosphere. Errors in T ± 1 °C.

Samples	T_onset_ (°C)	T_peak_ (°C)
pp-PLA	336	363
po-PLA	336	361
ha-PLA	318	350

**Table 5:** Young’s modulus (E), tensile strength (σ_s_) and strain at break of PLA film, po-PLA and ha-PLA electrospun fibers.

Sample	E (GPa)	σ_s_ (MPa)	Strain at Break (%)
PLA film	1.6 ± 0.3	25 ± 2	75 ± 9
po-PLA	0.34 ± 0.04	8 ± 1	31 ± 2
ha-PLA	1.0 ± 0.2	22 ± 5	27 ± 5
random-PLA	0.15 ± 0.05	2.0 ± 0.8	3 ± 2

## Data Availability

The data presented in this study are available on request from the corresponding author.
